# Fish biodiversity patterns of a mesophotic-to-subphotic artificial reef complex and comparisons with natural substrates

**DOI:** 10.1371/journal.pone.0231668

**Published:** 2020-04-24

**Authors:** Stuart T. Jones, Jacob M. Asher, Raymond C. Boland, Brian K. Kanenaka, Kevin C. Weng

**Affiliations:** 1 College of William & Mary, Williamsburg, VA, United States of America; 2 Joint Institute for Marine and Atmospheric Research, University of Hawaii at Manoa, Honolulu, HI, United States of America; 3 Ecosystem Sciences Division, Pacific Islands Fisheries Science Center, National Oceanic and Atmospheric Administration, Honolulu, HI, United States of America; 4 Department of Land and Natural Resources, State of Hawaii, Honolulu, HI, United States of America; 5 Virginia Institute of Marine Science, College of William & Mary, Gloucester Point, VA, United States of America; Florida Institute of Technology, UNITED STATES

## Abstract

Artificial reefs act as high-rugosity habitats and are often deployed to enhance fishing; however, the effects of man-made features on fish communities can be unpredictable and are poorly understood in deeper waters. In this study, we used a submersible to describe a deep-water artificial reef complex (93–245 m) off of Ewa Beach, Oahu, Hawaii, USA, and evaluated possible conservation and/or fisheries-related contributions. Sixty-eight species were recorded, with larger features supporting greater diversity of species. Species composition changed strongly with depth and a faunal break was detected from 113–137 m. While the features supported diverse fish communities, they were not similar to those on natural substrates, and were numerically dominated by only two species, *Lutjanis kasmira* and *Chromis verater*. Depth-generalist and endemic species were present at levels comparable to natural substrates, but were less abundant and species-rich than at biogenic *Leptoseris* reefs at similar depths. While the non-native *L*. *kasmira* was highly abundant, its presence and abundance were not associated with discernable changes in the fish community, and was not present deeper than 120 m. Finally, five species of commercially- and recreationally-important ‘Deep 7’ fisheries species were also observed, but the artificial reef complex was mostly too shallow to provide meaningful benefits.

## Introduction

Artificial reefs have historically been deployed to generate high-rugosity habitats, particularly in areas where reef-building corals have become degraded [1,2]. When well-designed, artificial reefs can enhance the rehabilitation of damaged ecosystems [3] and serve similar ecological functions as natural reefs [4]. Larger artificial reefs with greater habitat complexity typically support greater richness and evenness of fish species, and artificial reef structure, scale, and placement collectively play an important role in determining their impact on fish communities [4–6]. However, most artificial reef research has been conducted in shallow, SCUBA-accessible depths (<40 m), and biodiversity patterns remain poorly defined for artificial reefs located in mesophotic (40–130) to subphotic (>130 m) depths.

In Hawaii, two large-scale mesophotic-to-subphotic complexes have been deployed to provide additional structure in a low-rugosity habitat, one off of Ewa Beach, Oahu in 1987–88 (the features examined here), and one off Penguin Bank, Molokai in 1985 [7]. The Ewa Beach complex spans a wide range of depths (90–183 m), which, in natural communities, are associated with substantial biotic and abiotic changes as available light decreases [8]. These include major shifts to benthic communities, namely decreases in total living cover and transitions from scleractinian corals and macroalgae to crustose coralline algae [9–12]. Additionally, prior research suggests potential faunal splits at depths where light becomes insufficient for photosynthesis [9,10,13], below which fish species compositions may become markedly different. In certain areas, one major mesophotic split in reef fish communities has been identified between 50–60 m [9,10]. In Hawaii, though, perhaps due to the exceptionally clear waters [11], there are strong indications that another principal transition occurs between 110–140 m [8,13,14]. It is not known how these changes might affect artificial reefs, given that their physical structure is not dependent on available light, or whether artificial communities may exhibit faunal breaks that resemble those on natural substrates.

While there is increasing interest in artificial reefs as potential habitat enhancements, the associated fish communities they create are not always comparable to natural community structures [1]. While studies in shallower depths (0–40 m) indicate that artificial reefs with similar biotic and abiotic characteristics to natural reefs can support similar species compositions [3,4,15,16], artificial reefs which do not physically resemble natural reefs [4] are often found to have unusual or divergent fish community compositions [5,17]. The purposefully-scuttled ships, dry-dock caissons, and other features used for the Ewa artificial reef are much higher in relief than the generally low-relief basaltic outcrops and biogenic reefs dominated by the genus *Leptoseris* that naturally occur at these depths in Hawaii [8]. The similarity between fish communities at these deep artificial reefs and those on surrounding natural features is therefore unclear.

Deeper mesophotic reefs may also serve as refuges for some shallow-water species, as shallow-water reefs (<40 m) become increasingly degraded over time by anthropogenic stressors [9,10,18–22]. A subset of shallow-water species are ‘depth-generalists’, and can also occupy mesophotic depths > 40 m [9,11,12,23]. In cases where shallow-water populations of these species are diminished, e.g. due to overfishing or mass coral bleaching, there may be sufficient vertical connectivity for mesophotic populations to replenish shallow-water communities [8,9,21,24]. As such, if artificial substrates host substantial populations of depth-generalist fishes, particularly those that are targeted for extraction or which are sensitive to environmental changes, they may have the potential to act as refuges in mesophotic depths.

In Hawaii, a group of demersal and bentho-pelagic fishes found on slopes between 100–400 m support the deep-water handline or ‘bottomfish’ fishery [13,28]. Of these, six species of native snappers (*Aphareus rutilans*, *Etelis carbunculus*, *Etelis coruscans*, *Pristipomoides filamentosus*, *Pristipomoides sieboldii*, *Pristipomoides zonatus*) and the endemic Hawaiian grouper (*Hyporthodus quernus*) are collectively known as the ‘Deep 7’ and are among the most prized commercial fishes in Hawaii [29]. As a group, these species have long lifespans, slow growth rates, high ages to maturity [28,30], and have been subject to spatial fishery closures, seasonal constraints, and catch limits [30–32]. However, although they are present as adults on a series of artificial reefs near Molokai [24], it is unclear how the ‘Deep 7’ responds to artificial structures located in other mesophotic-to-subphotic locations around Hawaii.

Additionally, the non-native Blueline snapper (*Lutjanus kasmira*), known locally as ta'ape, may also occupy artificial reefs in Hawaii and is speculated among fishermen to compete with commercially and recreationally important native fishes [31,32]. However, prior research has not revealed any direct competition between *L*. *kasmira* and native snappers [33–35], though competition at the larval and juvenile stages has not been assessed. While conspicuous at several shallower artificial reefs around Oahu in SCUBA-accessible depths, the abundance of *L*. *kasmira* on mesophotic-to-subphotic artificial reefs, and any associated influence or impacts on surrounding reef or bottomfish communities, remain unexamined.

Finally, as one of the most isolated archipelagos in the world, Hawaii hosts high levels of reef fish endemism on both shallow and mesophotic coral ecosystems. Around 20% of species on shallow reefs in Hawaii are endemic [25], while studies in the Northwestern Hawaiian Islands (NWHI) indicate even higher endemism levels (46%) at mesophotic depths [26], and observations at certain reefs have recorded endemism levels up to 100% [27]. It is unknown if comparable endemism levels occur on deeper artificial reefs.

In this paper we focus on the following key questions:

1. What are the fish species richness and community composition at artificial reef features? Do features differ in the species they support? If so, are differences related to feature size and/or depth?
2. Are fish communities at artificial features similar to those on natural substrates at comparable depths?
3. Do artificial features host substantial numbers of depth-generalist species? Do artificial fish communities resemble those on shallower natural substrates?
4. Were species of interest (i.e. endemic, ‘Deep 7’, *L*. *kasmira*) present at the artificial features, and to what extent?

## Methods

### Study site

**Fig 1:**
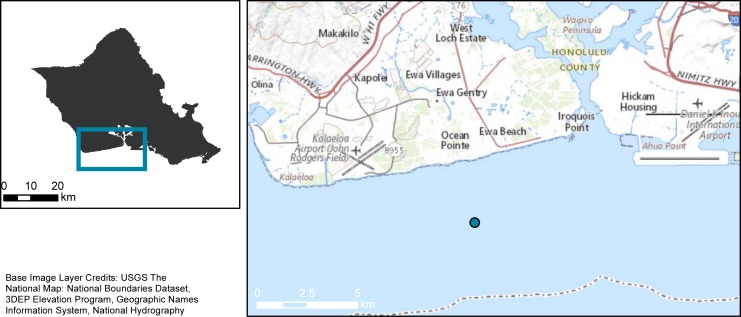
Map of the island of Oahu, Hawaii. The location of the artificial reef complex is depicted in blue. Figure by Tomoko Acoba (NOAA).

In total, nine underwater features at the Ewa Beach artificial reef complex were surveyed during HURL submersible dives 355, 356, and 357 on August 10–12, 1998. The Ewa Beach complex is composed of purposefully-scuttled vessels, caissons, and debris located on a mesophotic-to-subphotic sand slope approximately 2.5 km off of southern Oahu (Fig 1) [21.28 N; 158.02 W]. The complex is about 12 hectares (ha) in size, and due to time and resource constraints, a full census of the area was not practical. As a result, the survey design targeted six known features based on information provided by the Hawaii Department of Land and Natural Resources. These six targeted features were located between 90–185 m depth, and were each surveyed three times, making up the main sample of this study. Three deeper features that lie downslope of the official artificial reef complex were surveyed once, but could not be located again, and therefore could not be replicated. As a result, data from these deeper features were used only for descriptive statistics (i.e. presence/abundance of endemic, depth generalist, ‘Deep 7’ species), and are not included in any analytical comparisons between features.

### Data collection

The Pisces V submersible of the Hawaii Undersea Research Lab (HURL) was used to conduct daytime visual dive surveys of the Ewa Beach artificial reef complex, following the methods of an earlier submersible survey at Penguin Banks, Molokai, Hawaii [7]. In summary, three people were present in the submersible during the survey dives: the pilot, facing in the direction of travel, and observers positioned to either side of the pilot. Circular transects were conducted by navigating the Pisces V around the perimeter of each target feature. As described, a ‘feature’ is defined as a single vessel or large object, or, in the case of the ‘Rubble’ feature, a close clustering of smaller objects. During each circular transect, a ground speed of about 0.5 m/s was maintained to allow time for detailed visual surveying. The observers directed video recordings and logged species and abundance to the lowest possible taxon. Video was later annotated to provide a final log of species occurrence and abundance. We recorded the total number of individuals of each species as the maximum number seen at any one time during a circular transect survey. Known as MaxN, or historically as N_Max_, mincount, Maxsna, or MaxNO in other works, this method, while conservative, remains the standard for measuring the relative abundance of insular reef fish, bottomfish, and/or pelagic fish collected from remote sensing methods [25–28].

To compare fish assemblages observed at the Ewa Beach artificial reef complex to those found on natural benthic substrates, we incorporated data from three additional research studies of natural mesophotic-to-subphotic reefs in Hawaii. Each study provided a subset of data on fish populations found on one of three common, naturally-occurring habitats: mesophotic-to-subphotic hard-substrate slopes (90–185 m), mesophotic biogenic reefs dominated by *Leptoseris* scleractinian corals (70–110 m), and shallow-to-mesophotic aggregate biogenic reefs (30–50 m). For hard substrate slopes, we used a subset of NOAA Bottom Camera (BotCam) bottomfish data collected between 2011 and 2013 for three depth ranges: 96–117 m, 135–142 m, and 177–190 m, which were all categorized as “hard substrate” slope habitat and corresponded to depths of the features in the Ewa Beach complex [29]. For *Leptoseris* habitat, we used a subset of submersible surveys conducted by Boland [30] of mesophotic reefs of the Maui-Nui area between 70–75 m and 90–110 m for “*Leptoseris*” substrate habitat (biogenic reefs predominately composed of *Lepstoseris* scleractinian corals). Finally, we used a subset of NOAA unbaited remote underwater stereo-video (RUVS) fish data at ~30 m and ~50 m which surveyed areas with >50% coverage of hard coral, which were all characterized as “aggregate” (Asher, unpublished data).

### Analysis

Fish community data were examined in four parts, corresponding to the four key questions. First, the artificial reefs were analyzed by themselves. Second, all artificial features were compared to naturally-occurring substrates at comparable depths (90–110 m, ~137 m, ~183 m). Third, the shallower (<120 m) artificial reef features were compared to shallower naturally-occurring substrates (30–110 m). Finally, the abundances of species of interest (endemic, ‘Deep 7’, introduced) were quantified across all the artificial features, and we noted where abundances were significantly different between artificial and natural substrates.

Feature length and depth were initially examined at the Ewa Beach artificial reef complex at the time of survey to corroborate estimates given at the time of installation. All features were roughly rectangular, so height and width were estimated from photographic images collected from the Pisces V submersible, with individual feature volume calculated assuming a block shape. However, untransformed volumetric estimates created an asymptotic curve when compared with diversity metrics, so feature volumes were logarithm-transformed to restore linearity, and to allow for comparisons between three-dimensional size and diversity. These transformed values are herein referred to as ‘log-feature size’.

For univariate comparisons, species presence and abundance data were summed cumulatively for each feature (excluding those that were only surveyed once) across all three submersible surveys, and used to quantify alpha and beta diversity using the R community ecology package ‘vegan’ [42]. Alpha diversity, defined as the biodiversity present at each individual feature, was quantified using both cumulative ‘total’ species richness and the Shannon-Weiner index, which incorporates differences in species evenness between features. Beta diversity, defined as the change in diversity across features, was quantified using the Bray-Curtis dissimilarity index, which was preferred throughout the paper as an asymmetric, quantitative index that is widely appropriate for species x observations-type data. Linear regressions were used for all univariate comparisons, and are noted in the results. A mixed-effects model was used in one instance, also noted, where a previous independent variable had already been shown to correlate significantly with the response variable. In this case, log-feature size and depth were both used simultaneously as explanatory variables for alpha diversity (in turn species richness and Shannon-Weiner index).

Prior to any additional analyses, the community dataset (species x observations) was transformed logarithmically, due to wide and highly non-normal variations in abundances between species. The community data set was then converted to a dissimilarity matrix (observations x observations) using the Bray-Curtis index. All three community comparisons (Key Questions 1–3) were visually explored non-parametrically using non-metric Multidimensional Scaling (nMDS) due to concerns about normality. For goodness-of-fit, the final stress value is reported within each nMDS plot along with the p-value of the Monte Carlo assessment. The artificial-only analysis (Key Question 1) was further explored using average-linkage hierarchical clustering, which produced the highest cophenetic coefficient of the hierarchical methods tested. Differences between natural and artificial fish communities (Questions 2–3) were tested for significance using ANOSIM as a non-parametric test of within-vs-between group variances.

## Results

### Deep artificial reefs and their fish communities

**Fig 2:**
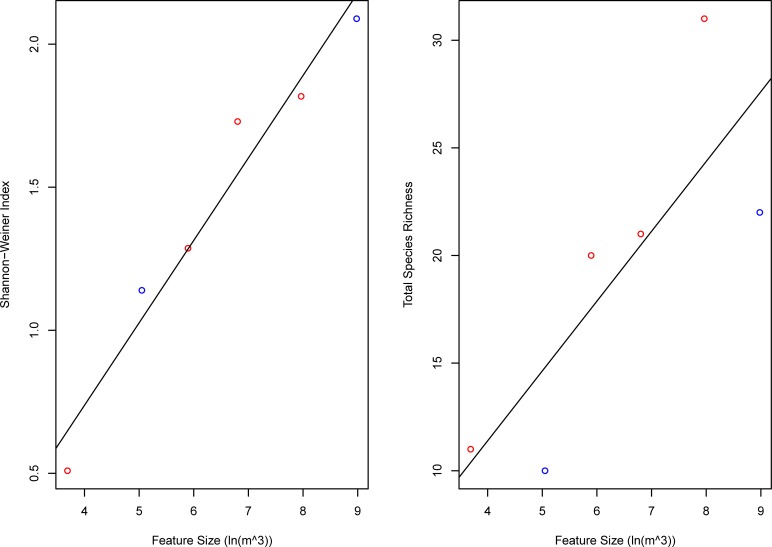
Alpha diversity vs. logarithm of volume and depth. Shannon-Weiner Index (A) and Species Richness (B) are plotted against logarithm of volume. To illustrate the effects of depth, features between 90–120 m are marked in red, and features between 135–185 m are marked in blue.

Larger features had higher total species richness (linear regression; m = 3.25, R^2^ = 0.65, p = 0.05) and Shannon-Weiner diversity values (linear regression; m = 0.29, R^2^ = 0.95, p = 0.0008) than smaller features (Fig 2), with the caveat that the circular transects were longer for larger features (Table 1). The number of species encountered at any individual feature was generally proportional to transect length, with the species encountered per transect-meter remaining even across the six main features (those which were surveyed three times) (Table 1). After accounting for the significant effects of log-feature size, depth retained a significant influence on species richness (generalized linear model; m = -0.14 species/m, p = 0.04), but not Shannon-Weiner diversity (generalized linear model; m = -0.0009, p = 0.71) (Fig 2). There were no interactive effects between depth and feature size for either metric of alpha diversity.

**Table 1:** Submersible site overview and summary statistics.

Feature Description	Depth (m)	Feature Dimensions (l,w,h) (m)	Transect Circum-ference (m)	Average Fish Abundance (per survey)	Fish Abundance (per m of Transect)	Total Species Richness	Species richness (per m of transect)	Proportionof Endemic Species	Shannon-Weiner Index
2nd Caisson	96	39,5,6	88	104±11	1.2	21	0.24	28%	1.73
Rubble	100	10,2,2	100	355±355	3.5	11	0.11	27%	1.14
Navy Barge	103	40,15,7	110	132±62	1.3	31	0.28	22%	1.817
1st Caisson	113	30,1,11	62	235±156	3.8	20	0.32	30%	1.286
Ketch	139	13,4,3	34	17.3±11.6	0.5	10	0.29	20%	0.509
Slabs*	175	9,3,1	24	45	1.9	6*	0.25	17%*	1.108*
Shipwreck	183	53,15,10	136	45.7±28	0.34	22	0.16	27%	2.089
Hard Bottom*	200	5,5,1	20	3	0.15	1*	0.05	0%*	0*
Machine Housing*	245	2,2,2	8	13	1.6	5*	0.63	20%*	1.415*

*Only surveyed once.

**Fig 3:**
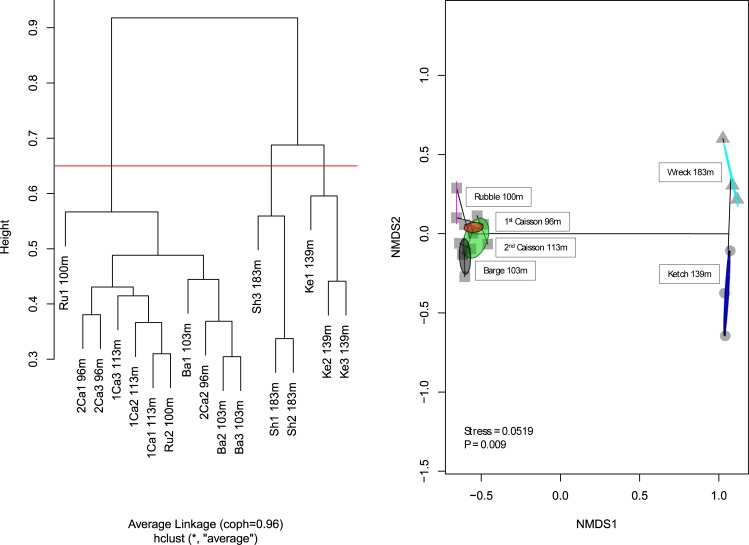
Biodiversity distribution across artificial features. Hierarchical clustering (Panel A) and nMDS (Panel B) both show significant differences between features either side of 130 m. At right, each observation is plotted in relation to the others based on the relative similarities between their observed species compositions, with observations grouped according to feature (colored ellipses), bridged hierarchically (black lines) according to Panel A, and designated according to cluster in Panel A at height h = 1 (gray symbols).

The difference in species composition between each possible pairing of the six features correlated strongly with each pair’s difference in depth (linear regression; R^2^ = 0.75, p<0.001), while the same was not true for feature size (linear regression; R^2^ = 0.067, p = 0.35). The nMDS ordination also suggested that species composition was strongly associated with depth (Fig 3B). Four features between 96 and 113 m (Rubble, Barge, 1^st^ and 2^nd^ Caissons) clustered together (Fig 3A), while the two deeper features between 139 and 183 m (Ketch and Shipwreck) were more distant. This was reflected in cluster analysis, with observations either side of 130 m grouping strictly together. Only 10 of 68 species were found on both sides of this divide, and ANOSIM showed that this grouping was highly significant (ANOSIM; R = 0.97, p = 0.0001), with the shallower features more similar to each other (ANOSIM; R = 0.43, p = 0.01) than the deeper features were (ANOSIM; R = 0.87, p = 0.1).

### Horizontal comparison with natural mesophotic and subphotic substrates

**Fig 4:**
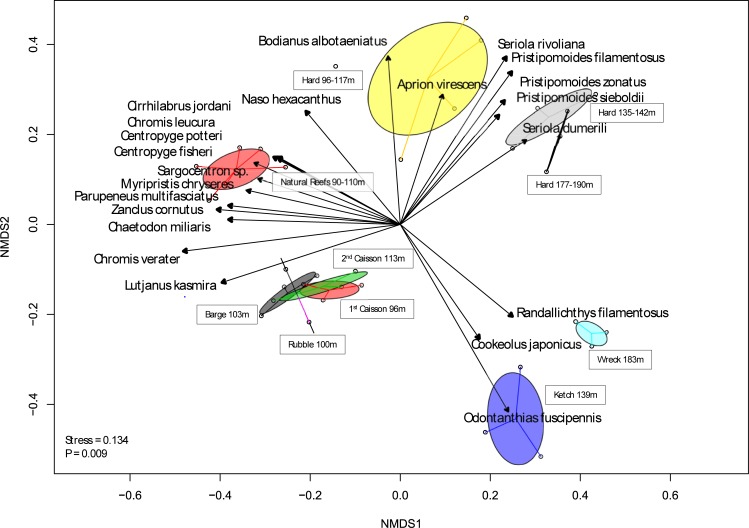
Horizontal comparison of artificial and natural substrates. nMDS analysis comparing all six artificial features with naturally-occurring habitat types at comparable depths. Observations are grouped (colored ellipses) by feature (artificial substrates) or depth (natural substrates). Loadings of individual species are shown (dark gray) for species with very significant (p<0.01) associations.

Fish communities associated with the Ewa artificial reef complex differed from those found on natural substrates at comparable depths i.e. *Leptoseris* reefs, and mesophotic-to-subphotic hard-substrate slope areas (Fig 4). These differences were significant both overall (ANOSIM; R = 0.32, p = 0.002) and at each depth bracket (ANOSIM: *Leptoseris* 90–110 m R = 0.55,p = 0.001; Hard slope 90–110 m R = 0.78,p = 0.001; 135 m R = 0.99,p = 0.01; 180 m R = 0.96, p = 0.018). As such, communities associated with artificial features (Caissons, Barge and Rubble) found at mesophotic depths were distant in ordination space from mesophotic *Leptoseris* reefs, and hard-substrate slope habitats as reported by Boland, and Merritt et al (2011), respectively [29–31]. Deeper mesophotic-to-subphotic artificial features (Ketch and Shipwreck) were also distant from BotCam hard-substrate slope data at their respective depths (139 m, 183 m) [30] (Fig 4).

### Vertical comparison and potential for depth-refuge

**Fig 5:**
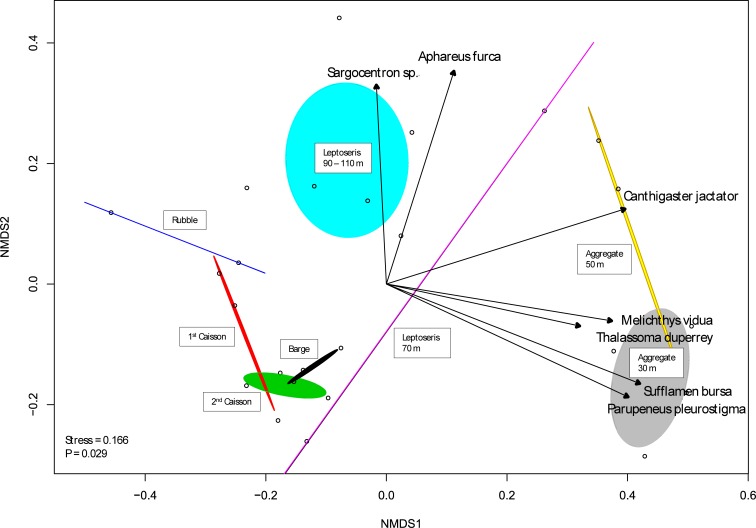
Vertical comparison of artifical and natural mesophotic reefs. nMDS analysis of the four shallower artificial features (93–117 m) compared to natural reefs at 90–110, 70, 50, and 30 m. Observations are grouped (colored ellipses) by feature (artificial substrates) or depth (natural substrates). Loadings of individual species are shown (dark gray) for species with very significant (p<0.01) associations.

Twenty-four species observed at the Ewa Beach complex also inhabit depths < 40 m as part of their documented range (Table 2) [32–34]. These represented 69% (24 of 35) of identifiable species observed from 93–113 m, and 79% (22 of 28) of those that were found exclusively in this depth range. These rates are slightly less than the rate of depth-generalism on *Leptoseris* reefs (84%, 49 of 58) and comparable to that of hard-substrate slope habitat (66%, 16 of 24). Fish communities at artificial features 90–110 m were, however, significantly different from those at biogenic reefs in similar or shallower depths (ANOSIM; R = 0.4, p = 0.001) (Fig 5).

**Table 2:** Inventory of species by depth stratum [32–34].

Species	Common <40 m	93–113 m	137–183 m	>200 m
*Acanthuridae* (unknown sp)		✓		
*Acanthurus dussumieri*	Yes	✓		
*Ammodytidae sp*.		✓		
*Antigonia sp*.		✓		
*Aphareus rutilans*		✓		
*Arothron hispidus*	Yes	✓		
*Bodianus bilunulatus*	Yes	✓		
*Caranx melampygus*	Yes	✓		
*Chaetodon fremblii*	Yes	✓		
*Chaetodon miliaris*	Yes	✓		
*Chaetodon multicinctus*	Yes	✓		
*Chaetodon tinkeri*		✓		
*Chromis verater*	Yes	✓		
*Coris flavovittata*		✓		
*Decapterus macarellus*	Yes	✓		
*Forcipiger longirostris*	Yes	✓		
*Genicanthus personatus*	Yes	✓		
*Gymnothorax steindachneri*	Yes	✓		
*Heniochus diphreutes*	Yes	✓		
*Holocentridae* (unknown sp)		✓		
*Lutjanus kasmira*	Yes	✓		
*Mulloidichthys vanicolensis*	Yes	✓		
*Myripristis berndti*	Yes	✓		
*Naso hexacanthus*	Yes	✓		
*Oplegnathus punctatus*		✓		
*Parupeneus insularis*	Yes	✓		
*Parupeneus multifasciatus*	Yes	✓		
*Parupeneus porphyreus*	Yes	✓		
*Pomacanthidae sp*.		✓		
*Pseudanthias bicolor*		✓		
*Pseudanthias fucinus*		✓		
*Sargocentron sp*.		✓		
*Scarus rubroviolaceus*	Yes	✓		
*Scarus sp*.	Yes	✓		
*Zanclus cornutus*	Yes	✓		
*Apolemichthys arcuatus*		✓	✓	
*Aulostomus chinensis*	Yes	✓	✓	
*Chromis struhsakeri*		✓	✓	
*Monacanthidae* (unknown sp)		✓	✓	
*Myripristis chryseres*		✓	✓	
*Odontanthias fuscipinnis*		✓	✓	
*Pristipomoides filamentosus*		✓	✓	
*Seriola dumerili*	Yes	✓	✓	
*Symphysanodon typus*		✓	✓	
*Roa modesta*		✓	✓	
*Bodianus sanguineus*	* *		✓	
*Congridae* (unknown sp)			✓	
*Cookeolus japonicus*			✓	
*Decapterus sp*.			✓	
*Hyporthodus quernus*			✓	
*Labridae* (unknown sp)			✓	
*Lutjanidae* (unknown sp)			✓	
*Muraenidae* sp.			✓	
*Ostorhinchus maculiferus*	Yes		✓	
*Priacanthidae* (unknown 1)			✓	
*Priacanthidae* (unknown 2)			✓	
*Pristiapogon kallopterus*	Yes		✓	
*Pristipomoides zonatus*			✓	
*Randallichthys filamentosus*			✓	
*Scorpaenidae* (unknown sp)			✓	
*Etelis carbunculus*			✓	✓
*Gymnothorax sp*.			✓	✓
*Odontanthias elizabethae*			✓	✓
*Antigonia sp*.				✓
*Dasyatidae sp*.				✓
*Liopropoma aurora*				✓
*Pontinus macrocephalus*				✓

### Commercial species (‘Deep 7’ Bottomfish)

Five commercially-targeted bottomfish species were found at the Ewa Beach artificial reef complex (Table 3), with species composition changing with increasing depth. *H*. *quernus*, *E*. *carbunculus*, *and P*. *zonatus* were found at depths greater than 170 m, while *P*. *filamentosus and A*. *rutilans* were encountered from 100–113 m. The most common ‘Deep 7’ species was *E*. *carbunculus*, which was found at all features in depths greater than 170 m.

**Table 3:** Abundances of the ‘Deep 7’ and *L*. *kasmira*. Features marked with an asterisk were only surveyed once.

Feature	2nd Caisson	Rubble	Navy Barge	1st Caisson	Ketch		Slabs*	Shipwreck	Hard Bottom*	Machine Housing*
Depth (m)	96	100	103	113	139	175		183	200	245
*Aphareus rutilans*	0	0	0	2	0	0		0	0	0
*Etelis carbunculus*	0	0	0	0	0	22		2	3	5
*Hyporthodus quernus*	0	0	0	0	0	0		3	0	0
*Pristipomoides filamentosus*	0	1	0	0	0	1		0	0	0
*Pristipomoides zonatus*	0	0	0	0	0	0		2	0	0
*Lutjanus kasmira*	69	900	80	270	0	0		0	0	0

### Introduced species *(Lutjanus kasmira)*

*L*. *kasmira* was highly abundant, but only at artificial features < 120 m (Table 4), and did not register any visible impacts to the overall fish community. *L*. *kasmira* abundance showed no relationship with total species richness (linear regression; R^2^ = 0.003, p = 0.37), but did show a significant relationship with the Shannon-Weiner index (linear regression; R^2^ = 0.56, p = 0.046). However, when *L*. *kasmira* and *C*. *verater*, another reef fish which co-occurred at high abundances with *L*. *kasmira*, were removed from Shannon-Weiner calculations, the correlation disappeared (linear regression; R^2^ = -0.17, p = 0.63). *L*. *kasmira* abundance also had no discernable effect on the species composition of other fishes (beta diversity) where it occurred (linear regression; R^2^ = -0.07, p = 0.46).

**Table 4:** Endemic species. Features marked with an asterisk were only surveyed once.

Feature	2nd Caisson	Rubble	Navy Barge	1^st^ Caisson	Ketch	Slabs*	Shipwreck	Hard Bottom*	Machine Housing*
Depth	96	100	103	113	139	175	183	200	245
Total Abundance									
*Apolemichthyes arcuatus*	2	2	2	4	0	0	0	0	0
*Bodianus sanguineus*	0	0	0	0	0	0	2	0	0
*Chaetodon fremblii*	0	0	2	0	0	0	0	0	0
*Chaetodon miliaris*	4	3	9	6	0	0	0	0	0
*Chaetodon multicinctus*	0	0	2	0	0	0	0	0	0
*Chromis struhsakeri*	0	0	0	1	0	0	3	0	0
*Chromis verater*	147	151	172	327	0	0	0	0	0
*Coris flavovittata*	0	0	1	0	0	0	0	0	0
*Gymnothorax steindachneri*	0	0	0	0	0	0	1	0	0
*Hyporthodus quernus*	0	0	0	0	0	0	3	0	0
*Liopropoma aurora*	0	0	0	0	0	1	0	0	0
*Odontanthias elizabethae*	0	0	0	0	0	0	8	0	5
*Odontanthias fuscipinnis*	11	0	5	2	48	0	34	0	0
*Ostorhinchus maculiferus*	0	0	0	0	1	0	0	0	0
*Parupeneus porphyrus*	1	0	0	0	0	0	0	0	0
*Pseudanthias fucinus*	26	0	0	20	0	0	0	0	0

### Endemism

We observed 16 species endemic to the Hawaiian Islands out of 68 total reported species (Table 4), with endemics representing 23.5 ± 4.3% of species encountered (Table 1). Endemic species composition also changed rapidly between 120–130 m, with ten endemic species noted in mesophotic depths and eight species in subphotic depths, but only two occurring across mesophotic and subphotic strata. Many endemic species were represented by only a small number of individuals (1–5 fish), though there were notable exceptions. In depths < 120 m the endemic *Chromis verater* was the second-most abundant species (147–327 individuals encountered) after the introduced *L*. *kasmira*, while > 130 m the endemic *Odontanthias fuscipinnis* was the most common fish encountered.

## Discussion

Larger artificial features supported higher alpha diversity when compared to smaller features (Fig 2); however, they did not support a greater density of species (Table 1). As a result, it is unclear whether a single large feature would support higher alpha diversity than several smaller ones with the same total volumetric size. In addition, larger features did not host significantly different species compositions than smaller ones (Fig 2), making the relative merits of larger vs. smaller objects further unclear.

The decline of species richness with increasing depth was weaker in our dataset than in previously published studies [35–37]. We observed a small decline in species richness, and no change in Shannon-Weiner diversity as depth increased (Fig 2). One explanation is that depth correlated with richness but not Shannon-Weiner diversity because the second metric considers species evenness. The highly abundant *L*. *kasmira* and *C*. *verater* were only found shallower than 120 m, driving down the evenness of these features in comparison to those located > 120 m. However, even with respect to species richness, depth had a relatively weak influence when compared with feature size (Fig 2B), and compared with the depth effect on natural mesophotic communities at similar depths [8,12,31]. Artificial reefs can host diverse communities even in deep water [5,10], and Moffitt et al. [7] similarly observed a weak effect of depth on species richness at the Penguin Bank artificial reef complex. In natural systems, benthic ecosystem engineers such as *Leptoseris* decline along with light at greater depths [10,11], whereas rugose artificial reef habitats remain independent of light. As such, artificial reefs may be somewhat resistant to the declines in alpha diversity that are typically observed in natural biogenic reefs with increasing depth.

Depth did primarily control variations in species composition, including an apparent faunal break from 113–139 m (Fig 3). This finding is consistent with Pyle et al.’s faunal break at 110–140 m and Weijerman et al’s. faunal break between mesophotic (30 – 129m) and upper subphotic zones (130 – 169m) at natural reefs in Hawaii, and Baldwin et al.’s zonal categorizations of Caribbean fish communities [8,14,38]. These faunal breaks have at times been speculated to be related to the depth at which light becomes insufficient for photosynthesis [9]. However, their existence on an artificial reef system suggests that they may not be wholly dependent on a loss of rugose habitat due to changes in benthic biota [11,12].

While the fish communities at the Ewa Beach artificial reef complex were diverse, they did not resemble those on natural substrates at similar depths (Fig 4). The *Leptoseris* reefs were distinguished by a number of taxonomically- and trophically-diverse species which were rare or absent from artificial reefs, including *Cirrhilabrus jordani*, *Centropyge potteri*, and *Myripristis chryseres*. These biogenic reefs also had greater percentages of endemic and depth-generalist species. The artificial features 90–110 m, meanwhile, were characterized mainly by only two species, *L*. *kasmira* and *C*. *verater* (Fig 4), which together accounted for nearly 90% of the total abundance of fish at those features. Interestingly, both of these species utilize reefs mainly for shelter [39,40], and venture out to feed in adjacent habitats, with *C*. *verater* feeding on plankton in the water column [24,40] and *L*. *kasmira* on infaunal invertebrates in sand flats [39,41]. Artificial reefs host fish communities which are most similar to natural ones when they physically resemble naturally-occurring substrates [4], and we propose that the Ewa Beach complex is mainly supporting fish species that have very general needs for their structural habitat.

At features surveyed from 93–113 m, the majority of identifiable species (24 of 35) were depth-generalists also found < 40 m. This was comparable to levels supported by natural substrates, both those examined by this study and at nearby Johnston Atoll [42], and previous research has also shown sufficient vertical genetic connectivity of *C*. *verater* (<40–113 m) to indicate possible refuge effects between shallow and mesophotic populations for this particular species [24]. However, the overall fish communities at the artificial reefs had very different compositions as those on shallower natural reefs, even more so than biogenic *Leptoseris* reefs at comparable depths (90–110 m) (Fig 5). As such, the depth refuge offered by these features may be less extensive than that offered by biogenic substrates, which themselves may be limited to depth generalists of certain trophic groups, such as planktivores and piscivores, or depth generalists with certain traits, such as a lunate tail and trailing fin filaments, which are proposed to be advantageous at greater depths or facilitate movement between depths [43]. Additionally, only four depth-generalist species were found on artificial features at subphotic depths (>135 m) (though several features in this depth strata were only surveyed once), so refuge-level effects may be limited to mesophotic features and only a subset of depth-generalist species.

While several ‘Deep 7’ species did occur at the Ewa Beach complex, the overall depth range of the site is mostly too shallow to provide meaningful benefits to five of these bottomfish species. The two species which can occupy these depths, *P*. *sieboldii* and *P*. *zonatus*, were much more abundant on natural hard-substrate slope habitat than on any of the artificial features (Fig 4). Generally, species that were most distinctive of the deeper hard-substrate slope habitat tended to be mobile carnivores (*Seriola* spp., *Pristipomoides* spp.), while those which loaded most strongly towards the subphotic artificial reefs were *Odontanthias fuscipinnus* and *Cookeolus japonicus*, smaller structure-associated species (Fig 4). Physical differences between these artificial features and hard-substrate slopes may be favoring more sedentary species over the roaming predators, including several ‘Deep 7’ species, that normally typify naturally-occurring habitats at these depths. However, this may also be due to differences in sampling bias between stationary cameras (BotCam) and a mobile submersible.

While *L*. *kasmira* was very abundant at the Ewa Beach complex, it was not associated with discernable changes to the surrounding fish community. This finding is consistent with publications on diet and foraging competition, which did not find strong evidence of competition between *L*. *kasmira* and native fishes at adult life stages [39,41,44]. While direct comparisons between *L*. *kasmira* and the Shannon-Weiner diversity index showed an initial, significant correlation, *L*. *kasmira* abundance had no detectable relationship with species richness. The correlation with Shannon-Weiner diversity, therefore, is primarily due to differences in species evenness. The high abundances of both *L*. *kasmira* and *C*. *verater*, which occupied the same depths and always co-occurred across artificial features, drove down evenness at features where *L*. *kasmira* was present, creating a misleading correlation. As mentioned above, *L*. *kasmira* prefers to shelter during daylight hours, dispersing at night to feed on benthic invertebrates found in unconsolidated sand and rubble [39]. As a result, it is unlikely to pose a danger to smaller fish or to compete with native snappers, which eat little of these benthic invertebrates in their adult life stages. Instead, the high abundances of *L*. *kasmira* simply seem to be enhanced by the same combination of depth, flat topography, and high relief that distinguish these features from natural substrates. Competition is still possible at other life stages, or through potential interactions other than diet overlap; however if competition were detectable at significant levels, we would expect that a high relative abundance of *L*. *kasmira* would result in corresponding changes to the abundances and/or diversity of surrounding reef fish communities. While the numerical dominance of *L*. *kasmira* indicates a major energy capture within the reef fish community, direct impacts on these artificial communities remain unclear and thus-far undetected.

Endemism at the Ewa artificial reef complex (23.5 ± 4.3%) was in between published levels for shallow-water reef fishes (20.5%) [45] and mesophotic fishes (27%) [8] in Hawaii. Pyle et al found considerably higher endemism levels (51%) for species found only deeper than 70 m than did this study. This discrepancy may be related to higher, localized endemism levels recorded around mesophotic *Leptoseris* reefs in the ʻAuʻau Channel and Maui-Nui region [8], and higher endemism levels where researchers targeted specific mesophotic habitats, such as hard-bottom, structurally-complex slopes and ledges [46,47]. Furthermore, technical divers may be better able to identify and survey fishes than observers in asubmersible. Taken together, these results suggest that the artificial reefs off of Ewa Beach support endemic biodiversity in a similar, albeit slightly lesser, capacity to nearby, less structurally-complex, natural substrates.

## Supporting information

S1 Data (XLSX)
